# GWAS of Second‐Generation Antihistamine Use: Locus Specificity and Highlighted Signalling Pathways

**DOI:** 10.1111/cea.70382

**Published:** 2026-07-02

**Authors:** M. Niemi, S. Ruotsalainen, E. Saarentaus, I. Vähätalo, F. FinnGen, I. Starskaia, T. Harju, M. Pelkonen, T. Feuth, P. Kauppi, J. Partanen, R. Lahesmaa, V. Hytönen, A. Palotie, H. Kankaanranta, T. Laitinen

**Affiliations:** ^1^ Institute for Molecular Medicine Finland FIMM, HiLIFE University of Helsinki Helsinki Finland; ^2^ Research Information Systems and Services, HUS IT Helsinki and Uusimaa Hospital District Helsinki Finland; ^3^ Computational Biology, Faculty of Medicine and Health Technology Tampere University Tampere Finland; ^4^ Department of Otorhinolaryngology – Head and Neck Surgery University of Helsinki and Helsinki University Hospital Helsinki Finland; ^5^ Tampere University Respiratory Research Group, Faculty of Medicine and Health Technology Tampere University Tampere Finland; ^6^ Turku Bioscience Centre University of Turku and Åbo Akademi University Turku Finland; ^7^ InFLAMES Research Flagship Center University of Turku Turku Finland; ^8^ Oulu University Hospital, The Wellbeing Services County of North Ostrobothnia and University of Oulu Oulu Finland; ^9^ Kuopio University Hospital Kuopio Finland; ^10^ Department of Pulmonary Diseases and Allergology Turku University Hospital and University of Turku Turku Finland; ^11^ Department of Pulmonary Diseases and Allergology, Heart and Lung Center Helsinki University Hospital and University of Helsinki Helsinki Finland; ^12^ Research and Development Finnish Red Cross Blood Service Helsinki Finland; ^13^ Institute of Biomedicine University of Turku Turku Finland; ^14^ Faculty of Medicine and Health Technology Tampere University Tampere Finland; ^15^ Fimlab Laboratories Tampere Finland; ^16^ Krefting Research Centre, Department of Internal Medicine and Clinical Nutrition, Institute of Medicine University of Gothenburg Gothenburg Sweden; ^17^ Department of Respiratory Medicine Seinäjoki Central Hospital Seinäjoki Finland

## Abstract

- Four of 67 loci were specific to antihistamine use; most overlapped inflammatory disease susceptibility.
- Genetic variation enriched in IL‐2 cytokine, JAK–STAT pathway genes shared by allergy and autoimmunity.

To the Editor,

1

Histamine is a central mediator of inflammatory responses released predominantly from mast cells and basophils following IgE‐dependent and independent activation [1]. Second‐generation H1‐antagonists (sgAHs) are first‐line therapy for allergic rhino‐conjunctivitis and urticaria and are widely used in asthma, atopic dermatitis and chronic rhinosinusitis [2]. Although sgAHs differ structurally, they share selective antagonism of the H1 receptor and a favourable safety profile compared with first‐generation antihistamines. Genome‐wide association studies (GWAS) of medication use may provide insights into disease mechanisms and pharmacologically responsive biological pathways beyond conventional diagnostic boundaries. We therefore performed a GWAS of long‐term, non‐seasonal sgAH use to identify genetic determinants associated with sustained treatment benefit and to distinguish loci reflecting medication use from those associated with underlying inflammatory diseases.

We analysed 392,000 participants from FinnGen Data Freeze 9 [3]. Cases were defined as individuals with > 10 systemic sgAH purchases, also outside pollen season, thereby enriching for long‐term and year‐round use (*N* = 39,804). Controls had no recorded sgAH purchases (*N* = 209,106), while individuals with 1–9 purchases were excluded. Medication use was identified from the nationwide drug purchase register (data available1995‐2021). Disease phenotypes were derived from linked nationwide healthcare registries. Genome‐wide association analyses of approximately 17 million variants were conducted using REGENIE v2.2.4 adjusted for age, sex, genotyping batch, ancestry principal components and relatedness. Fine‐mapping, HLA imputation and pathway analyses used SuSiE, HIBAG and MAGMA, respectively.

Cases had a mean age of 58 years, mean follow‐up of 20.4 years and 28.5 sgAH purchases; cetirizine was the most frequently used drug. Cases were more often female than controls (73.5% vs. 46.7%) and had as expected substantially higher prevalence of inflammatory mucosal and skin diseases, including asthma, allergic or vasomotor rhinitis, chronic sinusitis, conjunctivitis, atopic dermatitis and urticaria (65.4% vs. 18.3% among controls, *p* < 2.2 × 10^−16^). Disease‐specific corticosteroids were used by almost everybody among cases (95.5% vs. 65.3% among controls, *p* < 2.0 × 10^−16^), further supporting chronic inflammatory disease burden.

GWAS identified 66 independent loci (100 credible sets) and the HLA region associated with sgAH use at genome‐wide significance (*p* < 10^−8^) (Figure 1). Two of the identified lead variants were highly Finnish‐enriched missense variants in *IL4RA* and *TESPA1*. Additional coding signals included lead variants in *IL6RA* and *C17orf58* and a premature stop codon in *FLG*. Structural prediction suggested the *TESPA1* R282C/R144C substitution may be damaging, whereas the *IL4RA* substitution appeared benign. Several additional missense variants in linkage disequilibrium with lead variants (*r* ^2^ > 0.6) were identified, including loci previously implicated in eosinophilic and atopic disease biology.

**FIGURE 1:**
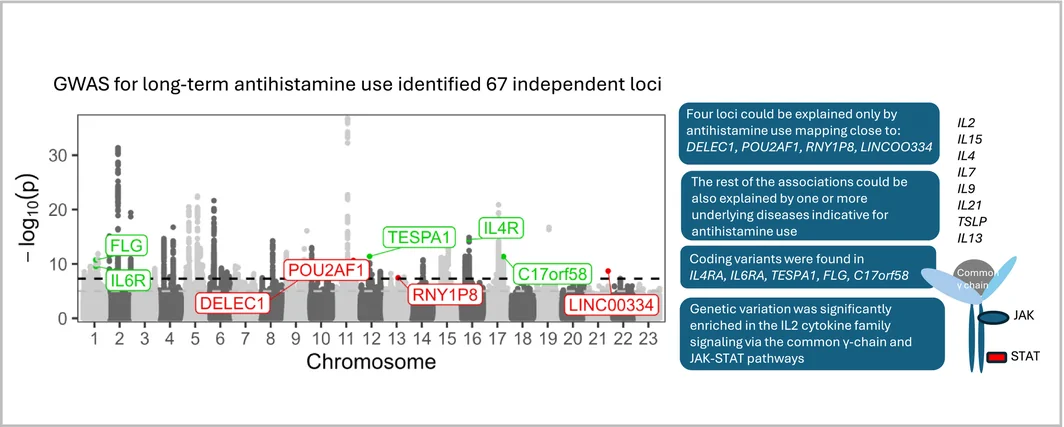
Genome‐wide association analysis of second‐generation antihistamine use highlighting locus specificity and associated signalling pathways.

HLA imputations showed associations to both class I and II genes. Two risk haplotypes corresponded to well‐established autoimmune‐associated haplotypes previously reported in FinnGen and other populations: C*07:01–B*08:01–DRB1*03:01–DQA1*05:01–DQB1*02:01 and DRB1*04:01/04:04–DRB4*01:03–DQA1*03:01–DQB1*03:02 [4]. Protective haplotypes included the most common Finnish haplotype and two rarer class II haplotypes without any known disease associations.

Gene‐based MAGMA analyses identified a total of 179 genes significantly associated with sgAH use. Pathway analysis showed enrichment that highlighted cytokine–cytokine receptor interactions, particularly IL‐2 family cytokine signalling through the Janus kinase/signal transducer and activator of transcription (JAK–STAT) pathway (*p* = 5.0 × 10^−9^) and shared the common γ‐chain (CD132) receptor subunit, a key regulator of lymphocyte development and function [5, 6] (Figure 1). In this pathway, 14 of 20 genes showed at least nominal association (*p* < 0.05) across 11 independent loci, suggesting convergence on a shared immunoregulatory mechanism. Enrichment of major histocompatibility complex class II genes was also observed, likely reflecting the single strong HLA signal.

Most loci overlapped with susceptibility to inflammatory diseases, with broadly consistent effect sizes across traits. Comparison of sgAH GWAS summary statistics with six overrepresented inflammatory conditions using MetABF suggested that most signals reflected shared disease susceptibility rather than sgAH responsiveness. However, four loci consistently demonstrated sgAH specificity, mapping near *DELEC1*, *POU2AF1*, *KLF5* and *LINC00334*. According to the GWAS Catalog, three of the loci appear largely independent of known disease associations. This may partly reflect incomplete diagnostic data but may also indicate biological mechanisms not captured by conventional disease classifications. The exception was rs13300483 near *DELEC1*, an eQTL for *TNFSF8*, previously associated with asthma outside FinnGen. Our GWAS also replicated eight of 14 previously reported sgAH loci [5, 7]. However, in those studies treatment indications were unavailable and most loci were established susceptibility loci for atopic or eosinophilic disease.

Our findings suggest that prolonged sgAH use is genetically linked to broad inflammatory and immunoregulatory pathways rather than to individual diagnostic entities. The enrichment of variants within IL‐2 family cytokine signalling pathways, together with the presence of autoimmune‐associated HLA haplotypes, supports the existence of shared biological mechanisms across allergic and immune‐mediated diseases [8]. These observations imply that genetic determinants of sgAH use may act through pathways that transcend conventional disease classifications and are therefore not fully captured by the diagnostic labels used to define treatment indications. Nevertheless, at least three loci appeared specific to sgAH use and were not associated with known disease susceptibility loci, raising the possibility that sustained benefit from antihistamines may reflect genetically mediated treatment responsiveness or patterns of medication utilisation rather than disease risk alone.

Limitations of the study include the lack of information on over‐the‐counter antihistamine use, incomplete diagnostic coding in early primary care and inability to reliably distinguish allergic from non‐allergic disease subtypes. Nevertheless, nationwide drug purchase and health registry data enabled long‐term characterization of treatment patterns. Individuals benefiting from sustained sgAH therapy may represent a biologically distinct subgroup relevant for future therapeutic targeting of shared cytokine‐mediated pathways [9].

## Author Contributions

M.N., H.K., R.L., I.V., J.P., V.H., A.P. and T.L. conceived and designed the study, drafted the original manuscript, reviewed and edited the manuscript, and obtained funding. M.N., S.R. and E.S. performed the statistical and bioinformatic analyses. T.H., M.P., I.S., T.F. and P.K. provided clinical and genetic expertise and critically revised the manuscript.

## Funding

We want to acknowledge the participants and investigators of the FinnGen study. The FinnGen project is funded by two grants from Business Finland (HUS 4685/31/2016 and UH 4386/31/2016) and the following industry partners: AbbVie Inc., AstraZeneca UK Ltd., Biogen MA Inc., Bristol Myers Squibb (and Celgene Corporation & Celgene International II Sàrl), Genentech Inc., Merck Sharp & Dohme LCC, Pfizer Inc., GlaxoSmithKline Intellectual Property Development Ltd., Sanofi US Services Inc., Maze Therapeutics Inc., Janssen Biotech Inc., Novartis Pharma AG, Boehringer Ingelheim International GmbH, Bayer AG and Alnylam Pharmaceuticals.

## Conflicts of Interest

H.K. reports personal fees for lectures and consulting from AstraZeneca, Boehringer‐Ingelheim, Chiesi Pharma, Covis Pharma, GSK, MSD, Orion Pharma and Sanofi outside the submitted work. T.F. reports lecture fees from GSK and consulting fees from Sanofi and AstraZeneca outside the submitted work.

## Data Availability

The data that support the findings of this study are openly available in Open Science Framework at https://doi.org/10.5281/zenodo.20342315 and GWAS summary statistics at FinnGen home page https://console.cloud.google.com/storage/browser/fg‐publication‐green/F_2022_010_20260512?pageState=(%22StorageObjectListTable%22:(%22f%22:%22%255B%255D%22)).
